# Artifact reduction in photoacoustic images by generating virtual dense array sensor from hemispheric sparse array sensor using deep learning

**DOI:** 10.1007/s10396-024-01413-3

**Published:** 2024-03-14

**Authors:** Makoto Yamakawa, Tsuyoshi Shiina

**Affiliations:** SIT Research Laboratories, Shibaura Institute of Technology, 3-7-5 Toyosu, Koto-ku, Tokyo, 135-8548 Japan

**Keywords:** Photoacoustic imaging, Sparse array sensor, Hemispherical array sensor, Deep learning, Artifact reduction

## Abstract

**Purpose:**

Vascular distribution is important information for diagnosing diseases and supporting surgery. Photoacoustic imaging is a technology that can image blood vessels noninvasively and with high resolution. In photoacoustic imaging, a hemispherical array sensor is especially suitable for measuring blood vessels running in various directions. However, as a hemispherical array sensor, a sparse array sensor is often used due to technical and cost issues, which causes artifacts in photoacoustic images. Therefore, in this study, we reduce these artifacts using deep learning technology to generate signals of virtual dense array sensors.

**Methods:**

Generating 2D virtual array sensor signals using a 3D convolutional neural network (CNN) requires huge computational costs and is impractical. Therefore, we installed virtual sensors between the real sensors along the spiral pattern in three different directions and used a 2D CNN to generate signals of the virtual sensors in each direction. Then we reconstructed a photoacoustic image using the signals from both the real sensors and the virtual sensors.

**Results:**

We evaluated the proposed method using simulation data and human palm measurement data. We found that these artifacts were significantly reduced in the images reconstructed using the proposed method, while the artifacts were strong in the images obtained only from the real sensor signals.

**Conclusion:**

Using the proposed method, we were able to significantly reduce artifacts, and as a result, it became possible to recognize deep blood vessels. In addition, the processing time of the proposed method was sufficiently applicable to clinical measurement.

**Supplementary Information:**

The online version contains supplementary material available at 10.1007/s10396-024-01413-3.

## Introduction

Vascular distribution is important information for diagnosing diseases, determining therapeutic effects, and supporting surgery. Therefore, there are various diagnostic devices for imaging blood vessel distribution. X-ray CT and MRI using contrast agents can image blood vessels as a 3D distribution with high contrast, but they require the use of a contrast agent and can only image relatively large blood vessels. Ultrasound Doppler imaging can image vascular distribution and blood flow velocity without using contrast agents, but it can only image relatively large blood vessels. In addition, optical coherence tomography angiography can image fine blood vessels without using contrast agents, but it can only measure blood vessels in very shallow areas. On the other hand, photoacoustic imaging, which has been put into practical use in recent years, is a technology that can image small blood vessels up to a depth of several centimeters with high contrast without using contrast agents [1–10]. Furthermore, photoacoustic imaging can also image the oxygen saturation distribution in blood using light of two or more wavelengths. However, photoacoustic imaging cannot image blood vessels unless light reaches the target, so it is difficult to image deep blood vessels compared to X-ray CT, MRI, and ultrasound Doppler imaging.

The photoacoustic effect is a phenomenon in which ultrasonic waves are generated from a light absorber when it is irradiated with pulsed light. The depth that can be imaged with photoacoustic imaging is determined by how deep the irradiated light reaches. Therefore, to image the living body as deeply as possible, it is preferable to use light that is less absorbed and scattered by living tissue, that is, near-infrared light. In fact, many photoacoustic imaging devices that target living tissues use near-infrared light.

In photoacoustic imaging, ultrasound generated from a light absorber is a very broadband signal. For example, it is known that ultrasonic waves generated from spherical absorbers have an N-shaped waveform [3]. Furthermore, ultrasonic waves generated from blood vessels also contain extremely wide frequency components. Therefore, an ultrasonic sensor with a wide reception frequency band is suitable for photoacoustic imaging. However, even when using a wideband sensor, there are limits to the frequency bands that can be received. Therefore, in photoacoustic imaging, signals before frequency bandwidth limitation are restored by inverse filtering using the frequency characteristics of the sensor, and image reconstruction is often performed using these signals.

Furthermore, the ideal arrangement of ultrasonic array sensors in photoacoustic imaging is a sensor arrangement that can measure the object from all directions. For example, a ring array sensor is ideal for 2D measurement. However, in 3D measurement, it is difficult to realize a sensor arrangement that completely covers the object. Therefore, array sensors with a finite aperture width are used, which causes a problem called the limited view problem, in which some blood vessels cannot be visualized. For example, a linear array sensor cannot receive signals from blood vessels running perpendicular to the sensor surface, and therefore cannot image them. Therefore, in 3D photoacoustic imaging, it is preferable to use a hemispherical array sensor arranged in a hemispherical shape around the object to measure the object with as wide an aperture angle as possible.

3D photoacoustic imaging requires a 2D array sensor. The 2D array sensor that can measure objects with a wide viewing angle requires an extremely large number of sensor elements. However, due to manufacturing and cost issues, sparse array sensors are often used in practice. Therefore, reconstruction artifacts occur due to sparse sensor density. One solution to reduce these artifacts is to measure the object by moving a sparse array sensor, which is virtually equivalent to measuring with many sensors. Although this solution has no problem when measuring a stationary target, it does not provide accurate images when measuring a moving target. Moreover, this solution is not applicable to observing target changes in real time.

Therefore, image reconstruction methods that reduce these artifact are needed when using sparse array sensors. In recent years, convolutional neural networks (CNN), a deep learning method, have been widely applied in the field of ultrasound measurement [11–24]. For example, research is being conducted on extracting blood vessels from photoacoustic images using CNN [20, 21]. However, artifacts caused by sparse array sensors appear curvilinear, so it is generally difficult to distinguish these artifacts from blood vessels. Therefore, we use a method that generates the received signals at the virtual sensors before image reconstruction. This method has been proposed for linear array sensors and ring array sensors [22–24]. In this method, virtual sensors are provided between adjacent real sensors, and signals of the virtual sensors are generated by interpolating from signals of the surrounding real sensors. However, since it is difficult to interpolate correctly with conventional interpolation methods (e.g., linear interpolation, spline interpolation), CNN is used. For 1D array sensors such as linear array sensors or ring array sensors, it is possible to train a 2D CNN for this interpolation using realistic computational resources (such as commercially available GPUs). In the case of 2D array sensors arranged in a grid on a plane, it is theoretically possible to generate virtual sensor signals using a 3D CNN. However, in this case, training a 3D CNN requires huge computational resources. In addition, in a hemispherical array sensor, the sensors are not arranged in a grid, so this method cannot be applied simply by expanding the dimensions.

Therefore, in this study, we focused on the regularity of the hemispherical array sensor arrangement and proposed a method to install virtual sensors between real sensors arranged in a 1D direction and perform this processing in three different directions. The proposed method can evenly arrange virtual sensors even in a hemispherical array sensor, making it possible to realize a virtual dense array sensor. Furthermore, since the proposed method uses three 2D CNNs, the processing in our method can be executed using realistic computational resources. In this study, we verified the effectiveness of the proposed method using simulation and human palm measurement data.

## Methods

### Hemispherical array sensor coordinates and virtual sensor settings

**Fig. 1:**
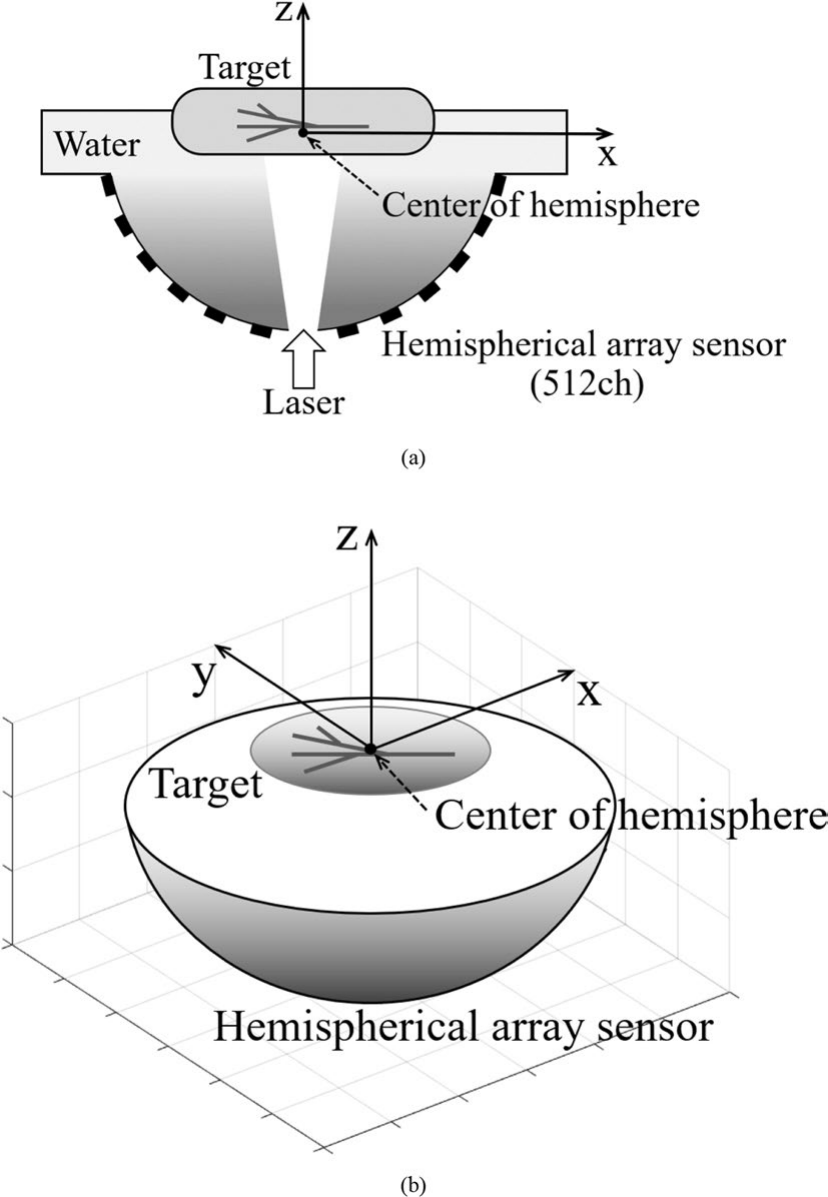
Schematic diagram of the measurement system using hemispherical array sensor. **a** Cross-section of the measurement system. **b** Overhead view of hemispherical array sensor and coordinate system used in this study

In a hemispherical array sensor, there is a method using the golden ratio and Fibonacci sequence to arrange the sensors evenly on a hemispherical surface [6, 7]. Each sensor coordinate (*x*_i_, *y*_i_, *z*_i_) obtained with this method is expressed by the following equations. In this study, we used coordinates with the center of the hemisphere as the origin, and the hemispherical array sensor was placed in the negative z-axis direction (see Fig. 1).

1 $${x}_{i}={r}_{i}{\text{cos}}\left\{\frac{2\pi i}{G}\right\}$$

2 $${y}_{i}={r}_{i}{\text{sin}}\left\{\frac{2\pi i}{G}\right\}$$

3 $${z}_{i}={z}_{1}+\left(i-1\right){d}_{z}$$

Here, *i* is the sensor number (*i* = 1, 2,…,*N*), *N* is the total number of sensors, *G* is the golden ratio ($G=\left(1+\sqrt{5}\right)/2$), *z*_1_ is the z coordinate of the first sensor (the sensor closest to the bottom of the hemisphere), and *d*_z_ is the sensor spacing in the z direction. When the radius of the hemisphere is *R*, *r*_i_ is expressed by the following equation.

4 $${r}_{i}=\sqrt{{R}^{2}-{{z}_{i}}^{2}}$$

**Fig. 2:**
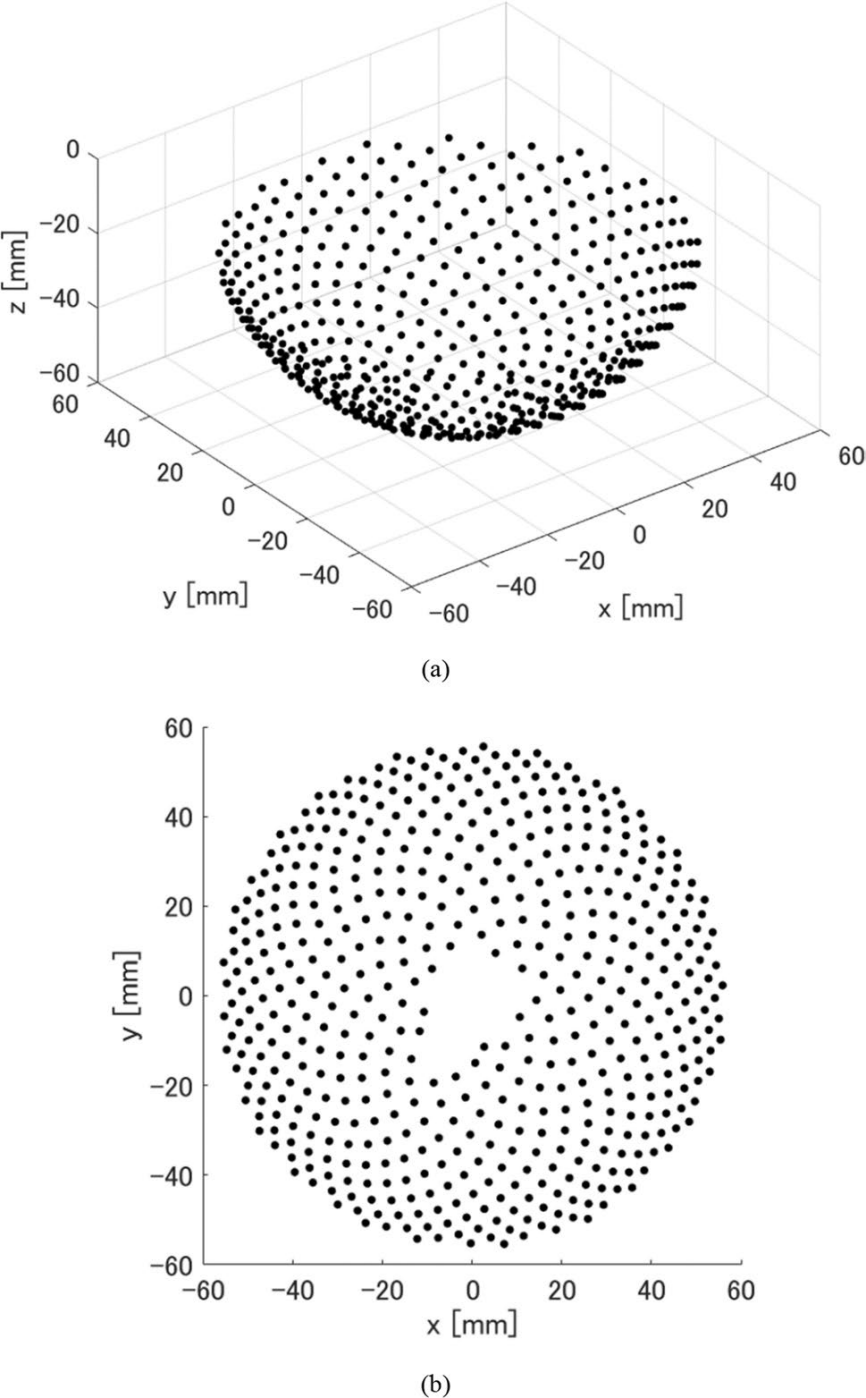
Arrangement of hemispherical array sensor. **a** Overhead view of hemispherical array sensor arrangement. **b** Sensor arrangement projected on the *x*–*y* plane

Figure 1 shows a schematic diagram of the measurement system and the coordinate system used in this study. Figure 2 shows the sensor arrangement of the 512ch hemispherical array sensor (Luxonus Inc., $R\cong$ 60 mm) used in this study. Figure 2a is an overhead view of the 3D sensor arrangement, and Fig. 2b shows the sensor arrangement projected on the *x*–*y* plane. Since there is a hole for laser irradiation at the center of the bottom of the hemisphere, no sensor is placed near the center of the bottom. Also, since there is a hole for water supply and drainage next to the laser irradiation hole, there are no sensors corresponding to numbers 7 and 20, but instead sensors are placed at the positions corresponding to numbers 513 and 514. Therefore, the total number of sensors is 512.

**Fig. 3:**
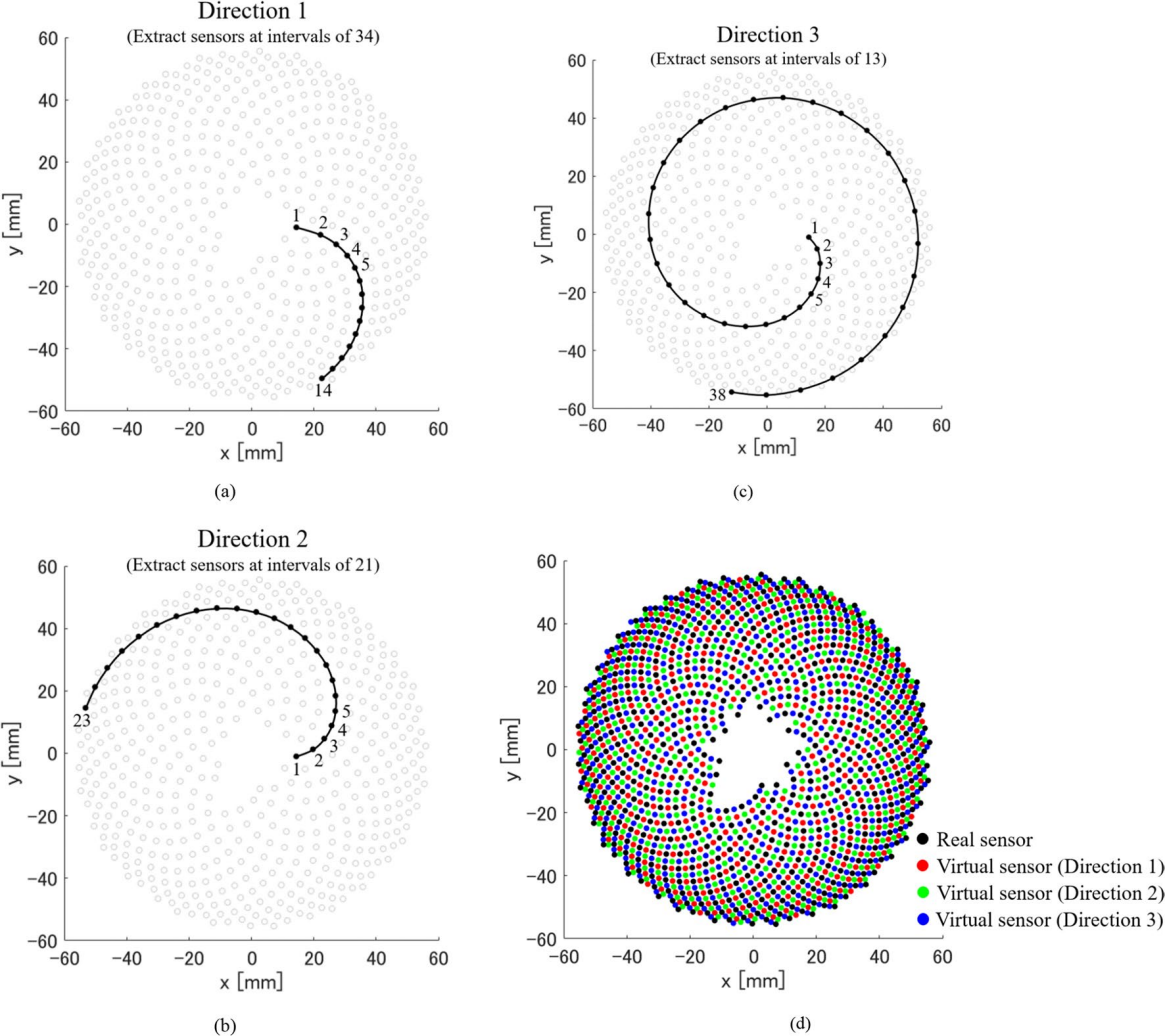
Three spiral patterns and virtual dense array sensor placement in hemispherical array sensor. **a** Spiral pattern in direction 1. **b** Spiral pattern in direction 2. **c** Spiral pattern in direction 3. **d** Virtual dense array sensor placement

When we look at the sensors arranged on a hemisphere based on the golden ratio and Fibonacci sequence, we can see that there are three spiral patterns (Fig. 3). These spiral patterns are obtained by extracting sensors at intervals of 34 (Fig. 3a), at intervals of 21 (Fig. 3b), and at intervals of 13 (Fig. 3c). Note that the spiral pattern extracted at intervals of 34 has the shortest sensor distance, and the spiral pattern extracted at intervals of 13 has the longest sensor distance. The numbers 34, 21, and 13 are part of the Fibonacci sequence. In this paper, we define the directions of these spiral patterns as direction 1 (at intervals of 34), direction 2 (at intervals of 21), and direction 3 (at intervals of 13), respectively. Note that these three directions correspond to the vertical, horizontal, and diagonal directions in a 2D grid sensor. Therefore, by providing virtual sensors between adjacent real sensors in each of the three directions, it is possible to set the sensors (real sensors and virtual sensors) almost evenly on the hemisphere (Fig. 3d). However, the number of real sensors extracted in each of these three directions is different, and even in the same direction, the number of real sensors extracted is different depending on the starting sensor number. Therefore, in this study, we used three CNNs corresponding to three directions, and determined the input size of each CNN based on the case where the number of real sensors extracted in each direction was the smallest. For that reason, some virtual sensors were not installed between the real sensors near the end of the spiral pattern (the outer periphery of the hemisphere). Note that the coordinates of the virtual sensor were determined by spline interpolation from the coordinates of the real sensor extracted in each direction and were taken as the coordinates of the center between adjacent real sensors. In addition, in the case of the 512ch hemispherical array sensor used in this study, the total number of sensors including real sensors and virtual sensors was 1897. In other words, the proposed method can increase the number of sensors to about four times that of real sensors.

### CNN for virtual sensor signal generation

**Fig. 4:**
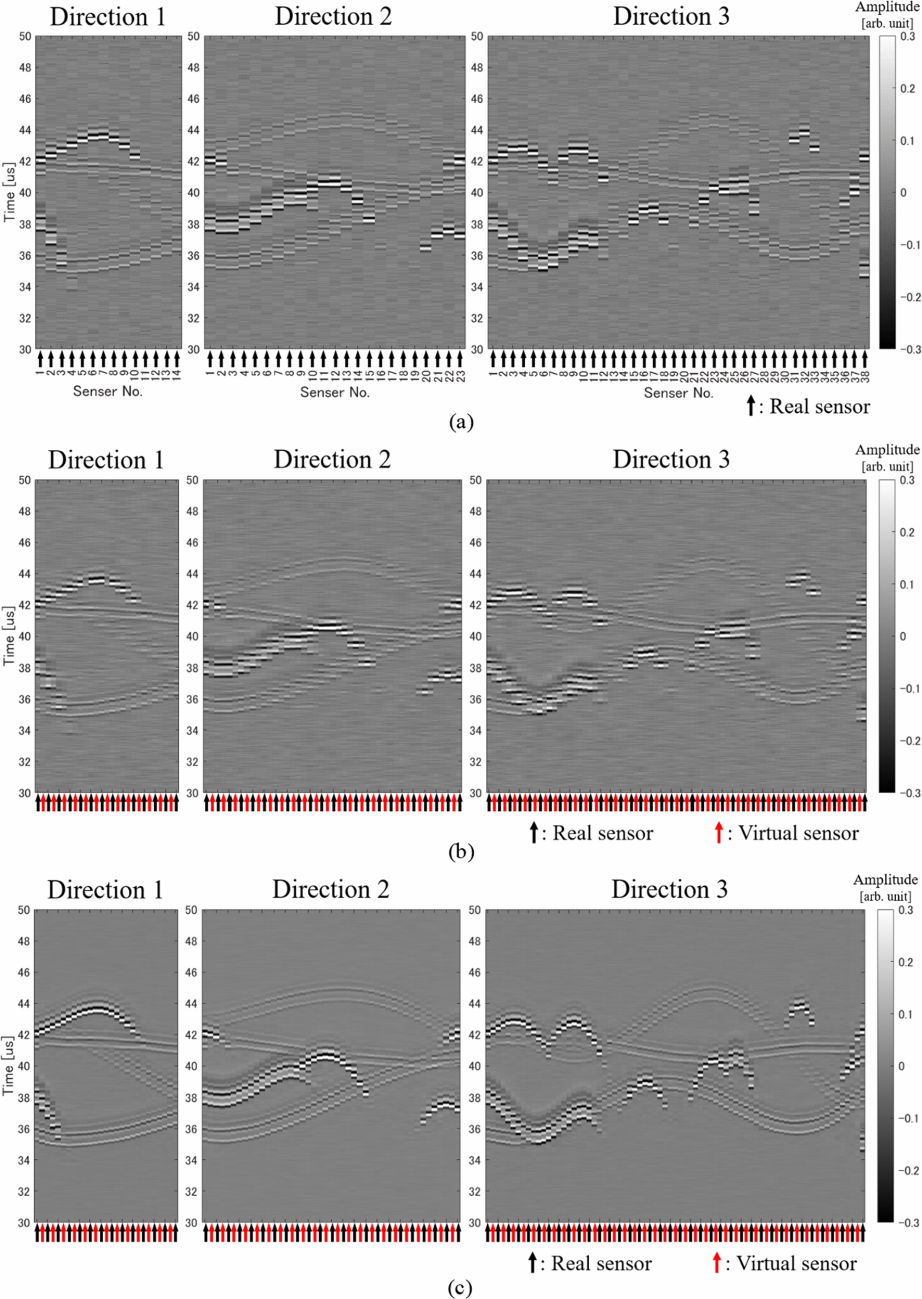

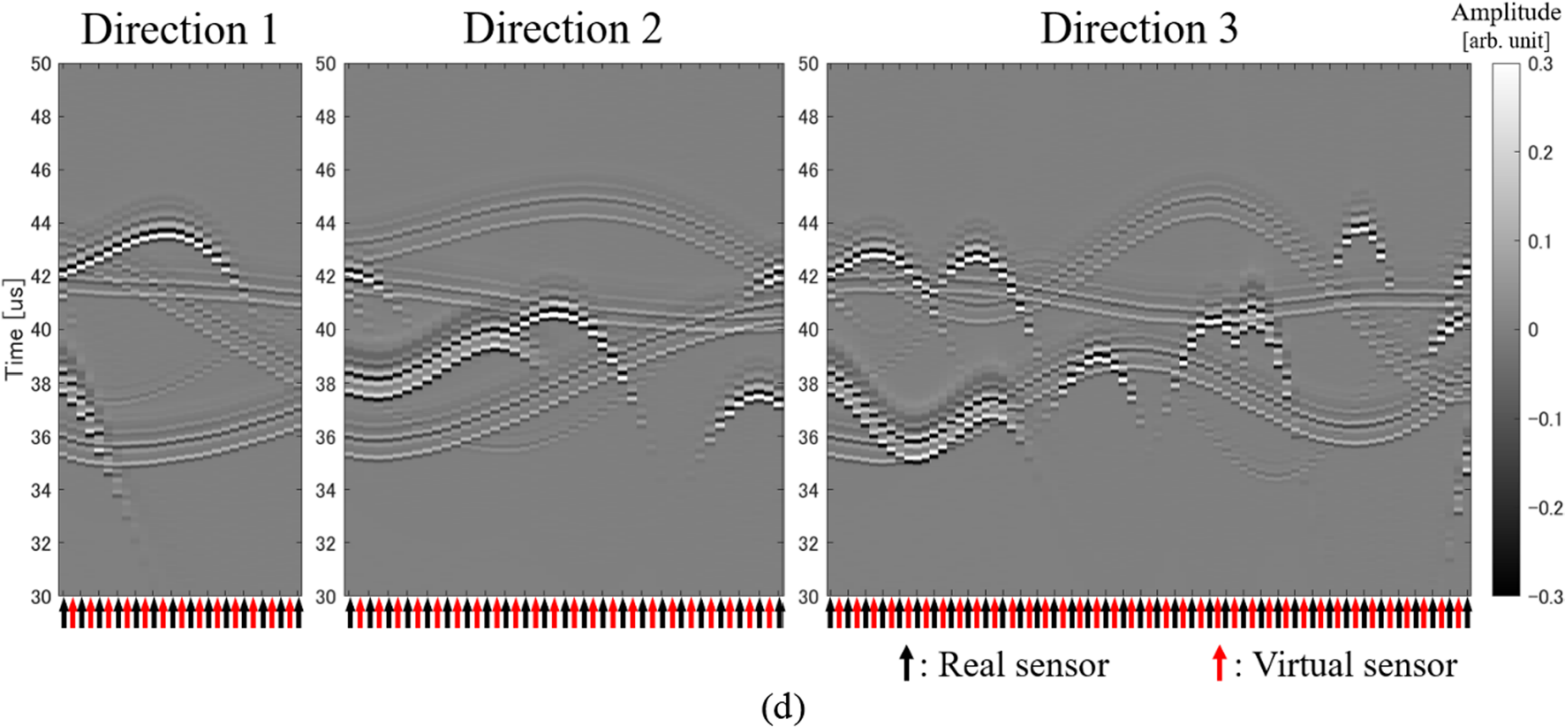
Examples of signal patterns along each direction. **a** Received signal pattern (real sensors only). **b** Linearly interpolated signal pattern (real sensors and virtual sensors). **c** CNN interpolated signal pattern (real sensors and virtual sensors). **d** Ideal signal pattern (real sensors and virtual sensors)

By extracting real sensors along the three directions mentioned in the previous section and arranging the received signals of the extracted sensors, a regular pattern is drawn according to the positional relationship between the light absorbers and the sensors (Fig. 4a). Therefore, the received signal at the virtual sensor can be estimated from this pattern. In other words, the positions of the virtual sensors are located between the real sensors lined up in each direction, so if we can successfully interpolate between the received signals of the real sensors, we can obtain the received signals of the virtual sensors. If there is only one absorber, it is possible to create virtual sensor signals by separately estimating the position and shape of the waveform from the absorber. However, in actual biological measurement, there are multiple absorbers and the signals from these absorbers overlap, so it is difficult to identify the waveform from a specific absorber. Therefore, in this study, we estimated the virtual sensor signals by interpolating the received signal pattern using a CNN. Note that the CNN used in this study does not identify the waveform from a specific absorber.

**Fig. 5:**
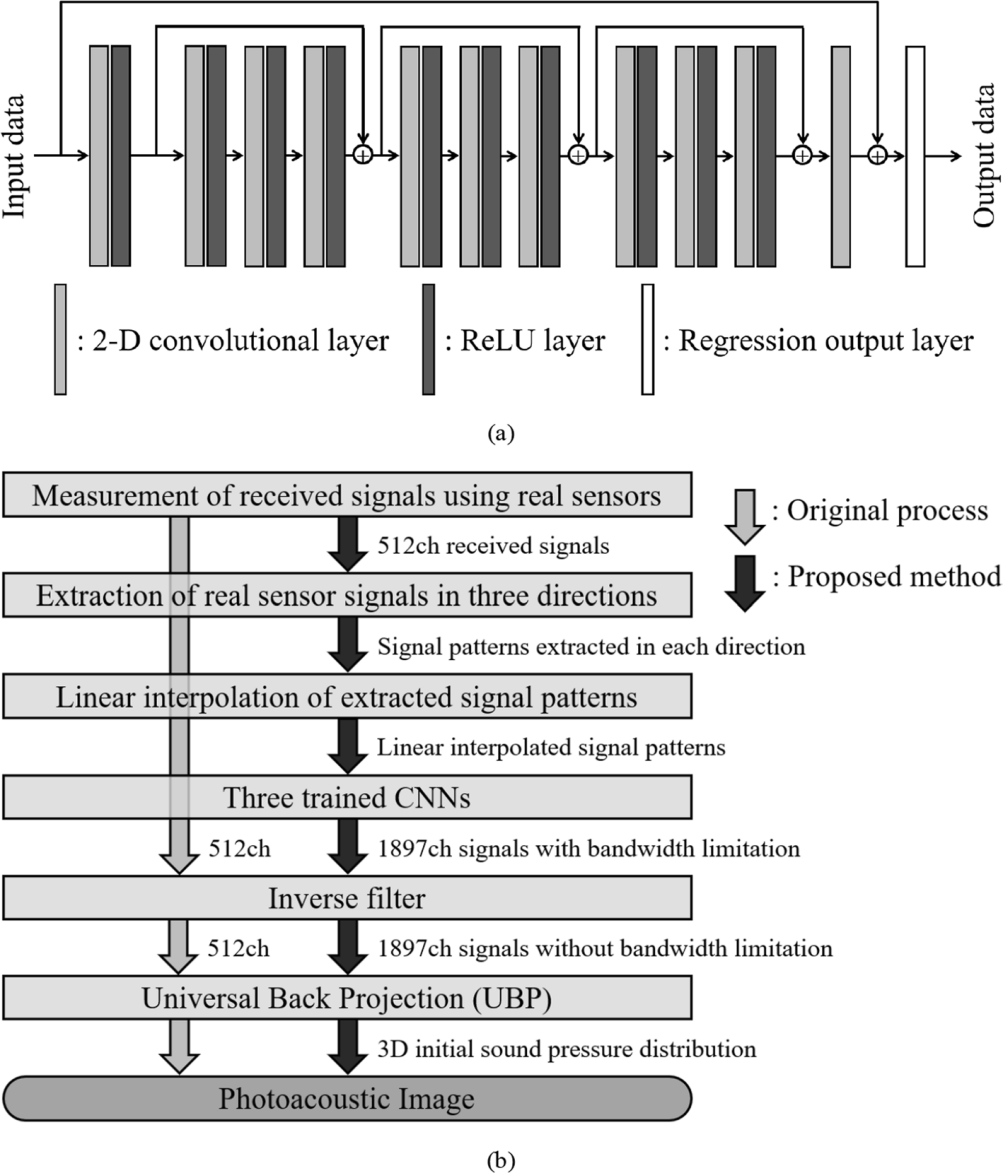
CNN used in this study and flowchart of photoacoustic image reconstruction using the proposed method. **a** CNN used in this study. **b** Flowchart of photoacoustic image reconstruction using the proposed method

There are various CNNs that can improve the resolution of images, but it is difficult to apply them to the data used in this study because the size of our data is larger than the typical image size handled by CNNs, and the size of each side is not a power of 2. Therefore, in this study, we used a CNN with a simple structure in which the data size did not change from input to output, as shown in Fig. 5a. In our CNN, we used multiple 2D convolutional layers, the LeRU function as the activation function, and a regression output layer that can output continuous values as the output layer. In addition, we used skip connections to solve the vanishing gradient problem and enable appropriate learning. In our CNN, we want the output to be data in which the virtual sensor signals are arranged between the real sensor signals, so the input data size should be the same as the output data size. Therefore, we used the data obtained by inserting linearly interpolated signals from the adjacent signals between the real sensor signals as input to our CNN. Here, we used linear interpolation only to make the input data size the same as the output data size, so performance regarding interpolation accuracy was not required at this stage.

We need three CNNs because the number of real sensors extracted in each of the three directions is different. However, we used one CNN for the same direction, and the CNN input size was determined based on the minimum number of sensors extracted in each direction. That is, in this study, the input and output size of the CNN in direction 1 was (14 real sensors + 13 virtual sensors) × 1792 samples, that in direction 2 was (23 real sensors + 22 virtual sensors) × 1792 samples, and that in direction 3 was (38 real sensors + 37 virtual sensors) × 1792 samples (see Fig. 3). Note that the number of samples in the time direction was determined to be as small as possible to cover the 3D reconstruction area. Here, the filter size was 7 × 77 (sensor direction × time direction) and the number of filters was 64, and these values were common to the three CNNs. An example output of our CNNs is shown in Fig. 4c. In addition, Fig. 4d shows the signal pattern when ideal signals are obtained from both the real sensors and the virtual sensors.

### Training of CNN

To train our CNN, we need input data and true output data. In this study, true received signals of virtual sensors were required for training of our CNNs. However, it is difficult to obtain virtual sensor signals in an actual system. Therefore, we created a training dataset through simulation. In our simulations, we used the sensor coordinates of the actual system and calculated virtual sensor coordinates from these coordinates. We created 1000 light absorber patterns consisting of multiple spherical absorbers and cylindrical absorbers randomly distributed with the parameters shown in Table 1, simulated the received signals at the real sensors and virtual sensors for each light absorber pattern and used them as training data. That is, the number of training data for the CNN in direction 1 was 34,000, that in direction 2 was 21,000, and that in direction 3 was 13,000. The sampling frequency of the received signal was 60 MHz, the same as the actual system, the sample length was 1792 samples, and the offset time from light irradiation to signal acquisition was 1500 samples. Note that the sample length and offset time were made as small as possible to cover the 3D reconstruction area.

**Table 1:** The conditions of light absorbers used for CNN training

Type of light absorber	Parameter	Value range
Spherical absorber	Number	1–4
	Diameter	0.2–1.2 mm
	Initial sound pressure	50–100 arb. units
	Center coordinates	Within a radius of 10 mm from the center of hemisphere
Short cylindrical absorber	Number	1–4
	Diameter	0.2–1.2 mm
	Length	10–20 mm
	Initial sound pressure	50–100 arb. units
	Center coordinates	Within a radius of 10 mm from the center of hemisphere
	Orientation of the cylinder in *x*–*y* plane	− 180 to 180 deg
	Angle with *x*–*y* plane	− 70 to 70 deg
Long cylindrical absorber	Number	1–4
	Diameter	0.2–1.2 mm
	Length	50 mm
	Initial sound pressure	50–100 arb. units
	Center coordinates	Within a radius of 10 mm from the center of hemisphere
	Orientation of the cylinder in *x*–*y* plane	− 180 to 180 deg
	Angle with *x*–*y* plane	− 5 to 5 deg

Next, we simulated the received signals before sensor bandwidth limitation at the real and virtual sensors for each light absorber pattern, and then applied bandwidth limitation to them based on the frequency characteristics (center frequency: 3.5 MHz, relative bandwidth: 90%) of the actual sensor. Finally, we added random noise to the received signals so that the signal-to-noise (*S*/*N*) ratio was 30 dB (i.e., − 30 dB noise relative to the signal amplitude after sensor bandwidth limitation from an absorber with an initial sound pressure of 100 arbitrary units at the center of the hemisphere). Based on the received signals from the real sensors obtained as described above, we used 2D data that were linearly interpolated from the received signals of the real sensors extracted in each direction as input data to our CNN. Furthermore, we trained the CNN using 2D data with the noise-free ideal received signals from the real sensors and virtual sensors as the ground-truth data. For CNN training, we used the resilient backpropagation (Rprop) method as the solver and set the learning rate to 10^–5^, the number of epochs to 50, and the mini-batch size to 50 (direction 1), 100 (direction 2), and 150 (direction 3). Since the CNN input/output data size differs depending on the direction and GPU memory is limited, the mini-batch size also differs depending on the direction.

### Processing during actual measurement

Figure 5b shows a flowchart of photoacoustic image reconstruction using the proposed method during actual measurement. First, we measure the received signals from the target with the real sensors, extract the received signals in each direction, and linearly interpolate the extracted signal pattern to create input data to the CNN. Then we process them with the CNN to obtain the output data (signals of real and virtual sensors). Next, we perform inverse filtering of the sensor frequency characteristics on the signals of real and virtual sensors to obtain signals before sensor bandwidth limitation. Here, this inverse filter processing is the process of applying a filter with characteristics that are the reciprocal of the sensor reception frequency characteristics and a low-pass filter to the received signals. Note that the purpose of using a low-pass filter is to not amplify high-frequency noise, and in this study, we used a low-pass filter with a cutoff frequency of 5 MHz. Finally, we reconstruct the 3D initial sound pressure distribution from those signals using a universal back-projection (UBP) algorithm [25]. Since the spatial resolution of the system used in this study was approximately 0.2 mm [6], we set the voxel size in reconstruction to 0.1 mm $\times$ 0.1 mm $\times$ 0.1 mm. Note that the UBP method is an image reconstruction method commonly used in photoacoustic imaging. The UBP method assumes that there is no refraction of ultrasound waves, and that attenuation is only diffusion attenuation, and performs image reconstruction based on the thermal diffusion equation and wave equation, like the filtered back-projection method used in CT image reconstruction. The UBP method is often used in other studies using a hemispherical array sensor [6–10, 26, 27]. However, there are also studies using the delay-and-sum method or delay-multiply-and-sum method [28] as the reconstruction method [29, 30].

The output data from the CNN also include signals corresponding to real sensors. However, although the signals corresponding to the real sensor output from the CNN are signals with reduced noise, the signal waveform also changes slightly (see Fig. S1 in the Electronic Supplementary). Since this change in signal waveform contains a smoothing effect, the reconstructed initial sound pressure may be underestimated. Therefore, we think that if the received signals have little noise, it is better to use the received signals themselves, and if the received signals have a lot of noise, it is better to use the signal output from the CNN. In this study, we wanted to compare the reconstruction results using only the real sensor signals and the reconstruction results by adding the virtual sensor signals to the real sensor signals, so we used the received signals themselves as the real sensor signals.

## Simulation experiment results

First, we conducted a simulation experiment to confirm the effectiveness of the proposed method. As a light absorber model for evaluation, we used a model in which three cylinders with a diameter of 1.0 mm, a length of 50 mm, and an initial sound pressure of 100 arbitrary units were lined up parallel to the y-axis. Here, the center coordinates of the three cylinders were (*x*, *y*, *z*) = (0 mm, − 5 mm, − 5 mm), (0 mm, 0 mm, 0 mm), and (0 mm, 5 mm, 5 mm), respectively. The coordinate arrangement and frequency characteristics of the hemispherical array sensors were the same as those used in the actual system and in creating training data. We also added random noise with the same amplitude as the training data (*S*/*N* ratio = 30 dB). In this paper, ‘noise in the received signal’ refers to only electrical noise (random noise), and ‘noise in the reconstructed distribution’ refers to noise due to electrical noise in the received signal and artifacts due to the reconstruction method.

**Fig. 6:**
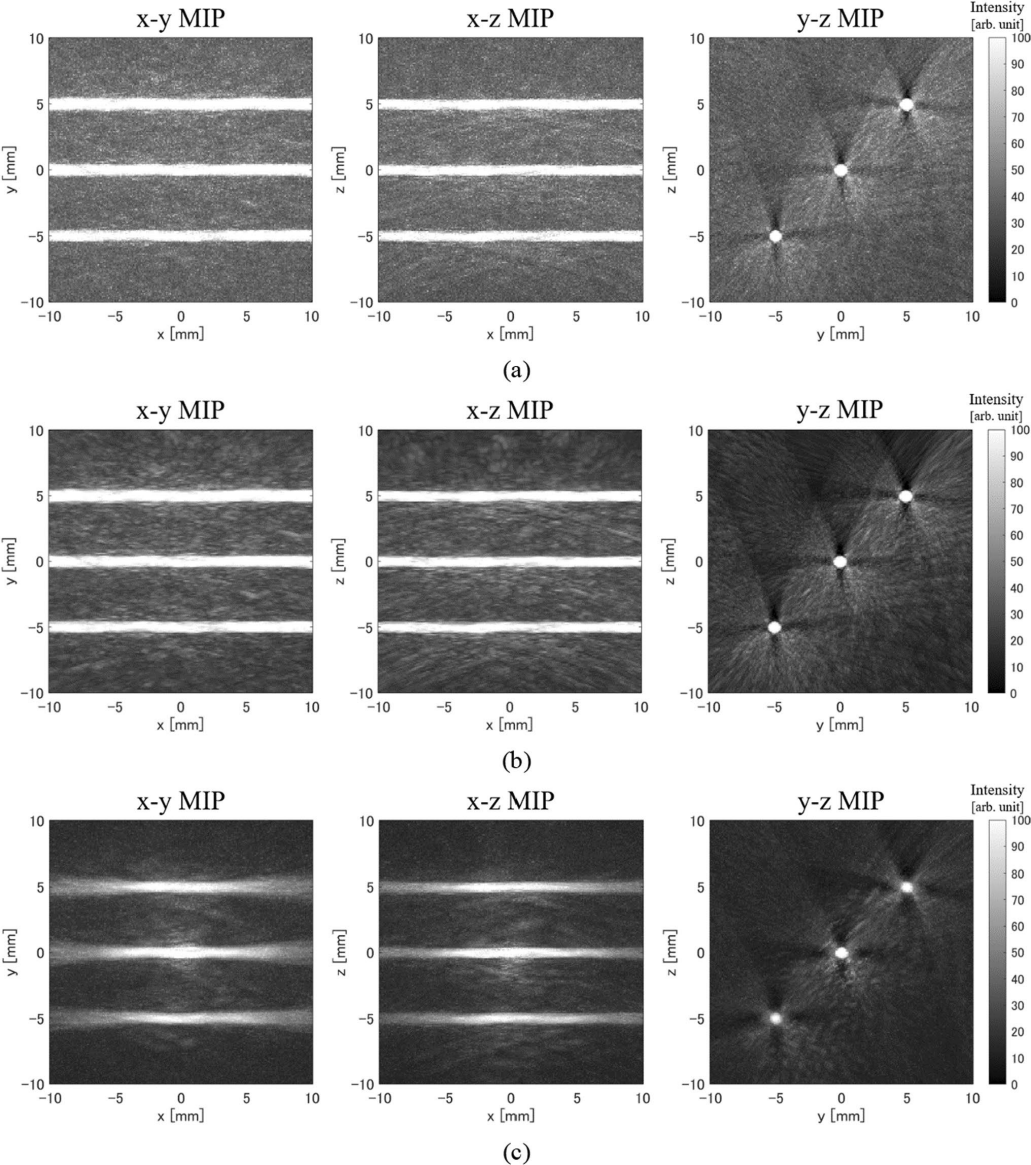

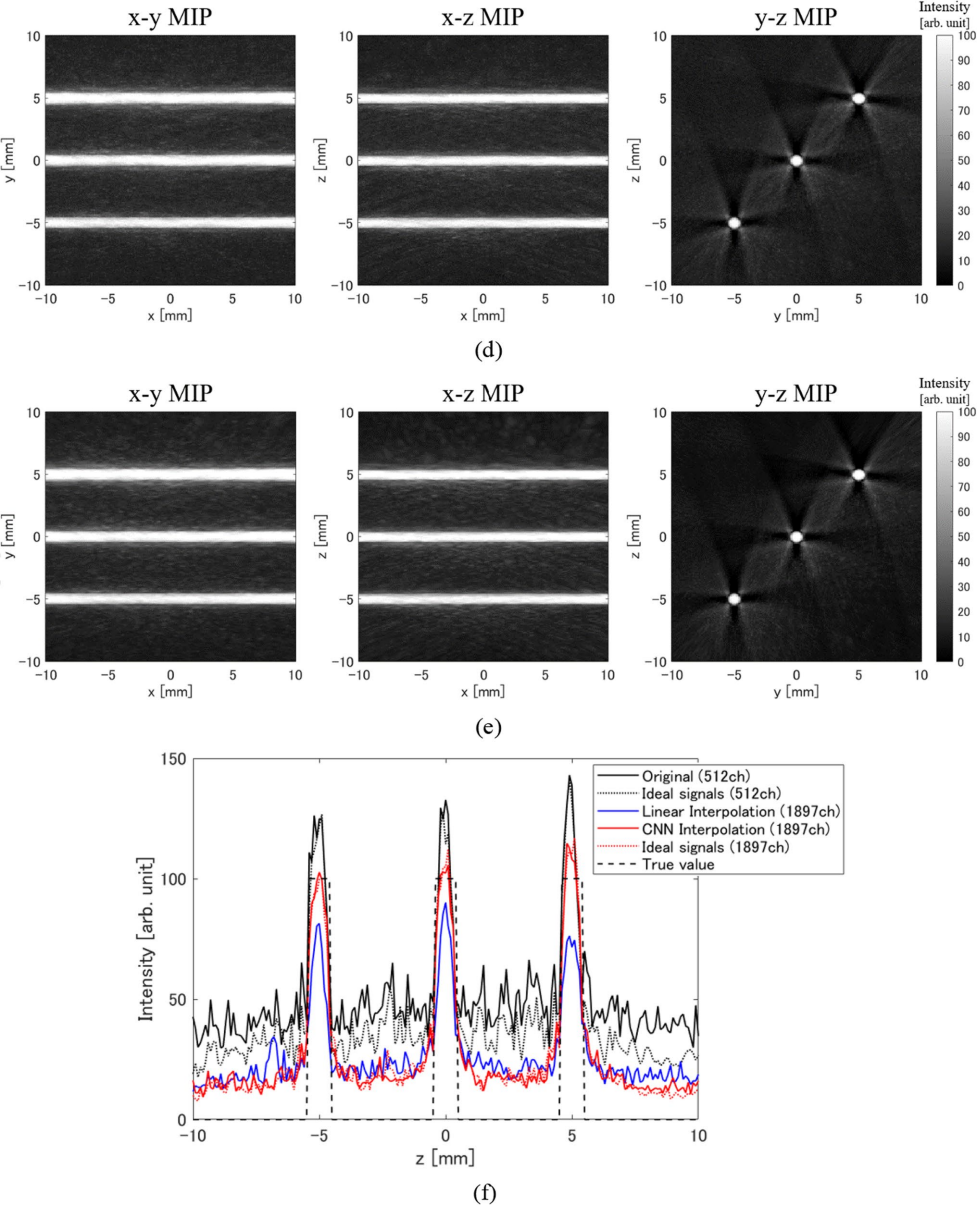
Simulation experiment results. **a** Reconstructed image using only real sensor signals. **b** Reconstructed image using only noise-free ideal real sensor signals. **c** Reconstructed image using real sensor signals and linearly interpolated virtual sensor signals. **d** Reconstructed image using real sensor signals and CNN interpolated virtual sensor signals. **e** Reconstructed image using noise-free ideal signals obtained from both real and virtual sensors. **f** Profile at *x* = 5 mm in *x*–*z* MIP images of each method

The simulation experiment results are as shown in Fig. 6. Figure 6 shows maximum intensity projection (MIP) images of the 3D reconstructed initial sound pressure distribution onto the *x*–*y* plane (left), *x*–*z* plane (center), and *y*–*z* plane (right), respectively. Figure 6a is the reconstructed image using only the real sensor signals, Fig. 6b is the result using only the noise-free ideal real sensor signals, Fig. 6c is the result when the virtual sensor signals were interpolated with linear interpolation, Fig. 6d is the result when the virtual sensor signals were generated using CNNs, and Fig. 6e is the result using the noise-free ideal signals at both the real and the virtual sensors. That is, the noise in the reconstructed images in Fig. 6b and e is only due to artifacts.

First, images reconstructed using the received signals only from the real sensors had strong artifacts because the sensor density was sparse (Fig. 6a). On the other hand, the reconstructed images when using CNN to interpolate the virtual sensor signals had reduced artifacts and increased absorber contrast (Fig. 6d). Moreover, the results of CNN interpolation were very close to the results when ideal received signals were obtained at both the real and the virtual sensors. Therefore, it is thought that CNNs can estimate signals close to the ideal signals in the virtual sensors. Note that the correlation coefficient between the signals estimated using the CNNs and the ideal signals at the virtual sensors was 0.933. This also indicates that the virtual sensor signals were estimated with high accuracy with the proposed method. The results of interpolating the virtual sensor signals using linear interpolation showed that the artifacts were reduced due to the increased number of sensors, but since the virtual sensor signals were incorrect, the absorber distribution was also incorrect (Fig. 6c). It is difficult to correctly interpolate virtual sensor signals using conventional interpolation methods (e.g., bi-cubic interpolation, spline interpolation) including linear interpolation. Therefore, in this paper, we showed the results using liner interpolation, which is one of the conventional interpolation methods and is also the input data to our CNN.

To quantitatively evaluate the results of the reconstructed 3D distribution, we calculated the S/N ratio using the mean intensity value in the light absorber as the signal amplitude and the standard deviation value of the background as the noise level. The *S*/*N* ratio results are shown in Table 2. This result confirms that the proposed method improves the *S*/*N* ratio by + 7.7 dB compared to the original result using only real sensor signals. On the other hand, the *S*/*N* ratio improved by + 6.1 dB by increasing the number of sensors under conditions using ideal signals. When using ideal signals, the noise in the reconstructed distribution is artifact only. Based on the results in Table 2, the main reason for improvement in S/N ratio with the proposed method is the reduction of artifacts.

**Table 2:** S/N ratio results in simulation experiments

Method	*S*/*N* ratio (dB)
Original (512 ch)	16.0
Ideal signal (512 ch)	18.7
Linear interpolation (1897 ch)	19.1
CNN interpolation (1897 ch)	23.7
Ideal signal (1897 ch)	24.8

## Results in human palm measurement

**Fig. 7:**
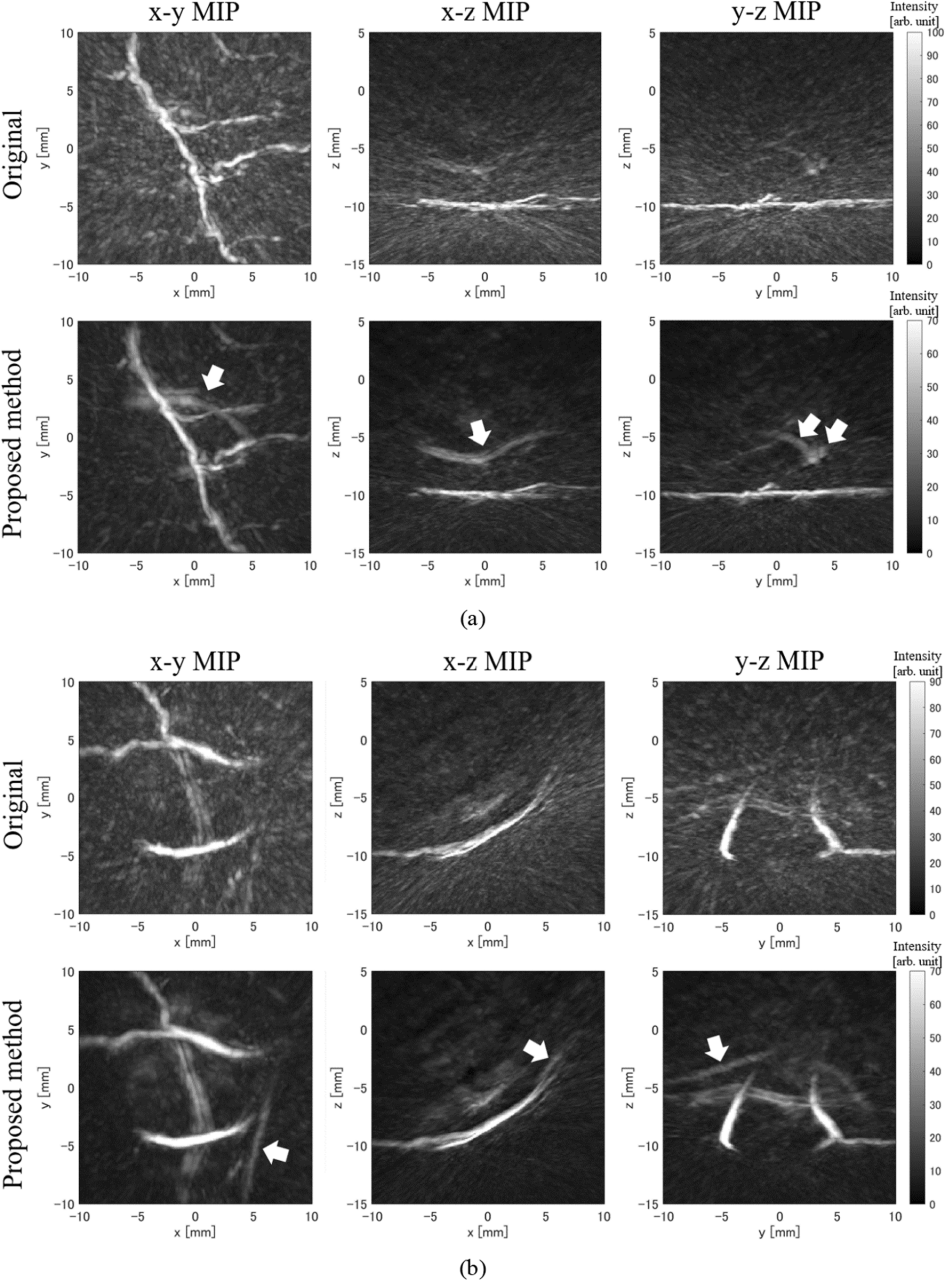

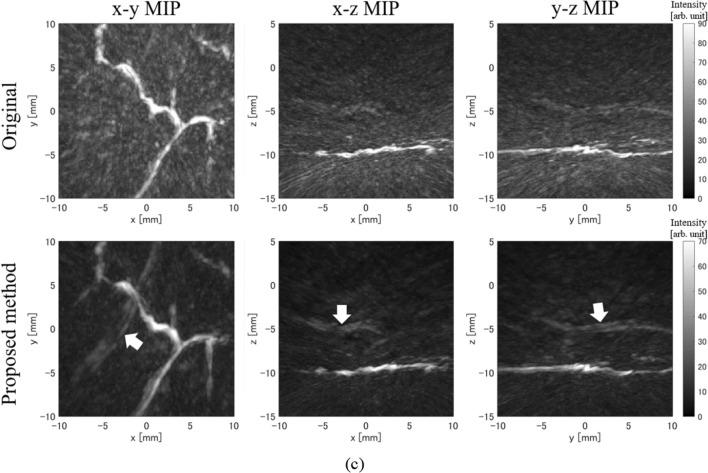
Human palm measurement results. The upper row shows images reconstructed from only real sensor signals, and the lower row shows images reconstructed using the proposed method. Arrows indicate deep blood vessels that are easier to recognize using the proposed method. **a** Example 1. **b** Example 2. **c** Example 3

Next, we used palm measurement data from healthy volunteers provided by Luxonus Inc. to confirm the effectiveness of the proposed method with real data. The data used in this study were measured using light with a wavelength of 797 nm and a hemispherical array sensor (center frequency: 3.5 MHz, relative bandwidth: 90%) with the sensor arrangement shown in Fig. 2. Figure 7 shows the results of measurements taken at three locations on the same palm. In all instances, we can see that the results of the proposed method show reduced artifacts compared to the original results of reconstruction from only the real sensor signals. Furthermore, deep blood vessels that were hidden by strong artifacts of superficial blood vessels in the original image became recognizable in the image obtained using the proposed method (arrows in Fig. 7).

To quantitatively evaluate these palm measurement results, we calculated the *S*/*N* ratio. However, since the correct blood vessel distribution cannot be determined from actual measurement data, we set the area with a threshold value of 40 or higher in the reconstruction distribution of the proposed method as a blood vessel area and set the area (− 15 mm ≤ *z* < − 11 mm) outside the palm as a background. Then we calculated the *S*/*N* ratio by dividing the average intensity of the blood vessel area (the blood vessel area identified with the proposed method was used even in the evaluation of the original results) by the standard deviation of the background. The *S*/*N* ratio results are shown in Table 3, and in all measurement results, the proposed method had a higher *S*/*N* ratio than the original results. The improvement of the *S*/*N* ratio yielded by the proposed method was also large in actual measurements, confirming that the proposed method is just as effective in the actual measurement data as in the simulation.

**Table 3:** S/N ratio results in human palm measurement

Method	*S*/*N* ratio of example 1 (dB)	*S*/*N* ratio of example 2 (dB)	*S*/*N* ratio of example 3 (dB)
Original (512 ch)	17.2	18.3	17.2
Proposed method (1897 ch)	22.8	25.8	22.7

## Discussion

First, the results of simulation experiments confirmed that the proposed method can significantly reduce artifacts caused by sparse sensor density, and the *S*/*N* ratio can be significantly improved to + 7.7 dB. Note that the artifacts caused by the sparse sensor density in the UBP method are like those caused by the small number of projections in the filtered back-projection method for X-ray CT. That is, as the number of sensors increases, the number of back projections from each sensor in the absorber region increases, whereas in regions other than the absorber, the back projections from each sensor do not overlap. Therefore, as the number of sensors increases, the value of the absorber region becomes relatively larger, and artifacts (back projections to regions other than the absorber) are reduced. Here, the method of interpolating virtual sensor signals using simple linear interpolation was also effective in reducing artifacts because the number of sensors is increasing. However, because the linear interpolation cannot create correct virtual sensor signals, the reconstructed distribution was not the correct distribution, and the error increased as the distance from the center of the hemisphere increased. This is thought to be because signals from near the center of the hemisphere arrive at all sensors almost simultaneously, so even the linear interpolation was able to interpolate the virtual sensor signals relatively accurately. However, for all virtual sensor signals, the correlation coefficient between the linearly interpolated signal and the ideal signal was 0.061.

In addition, the quantitative evaluation results of simulation experiments showed that the *S*/*N* ratio of the proposed method was almost the same as the result using ideal sensor signals, and it was confirmed that the proposed method was able to interpolate the virtual sensor signals accurately for the most part. However, the *S*/*N* ratio of the proposed method was slightly lower than the result using ideal virtual sensor signals. One of the reasons for this is thought to be that the virtual sensor signals yielded by the proposed method were slightly different from the ideal signals (see Fig. S2 in the electronic supplementary). That is, the virtual sensor signals yielded by the proposed method had a slightly smaller amplitude than the ideal signals and were also slightly shifted in the time direction, so it is thought that the value of the absorber region was estimated to be slightly smaller. Another reason is that the proposed method uses slightly noisy received signals in the reconstruction, whereas the result from ideal signals uses noise-free signals (at both real and virtual sensor signals).

In the simulation experiments, the initial sound pressure of the absorber reconstructed using only real sensor signals was higher than the set value (true value), while the initial sound pressure reconstructed using the proposed method was close to the set value. Similar results were obtained when ideal signals were used. Therefore, this is mainly caused by the difference in the number of sensors used for reconstruction. In other words, it is considered that there were not enough projections to perform correct reconstruction even in the absorber region using only the signals from the real sensor.

Regarding the palm measurement results, it was confirmed that artifacts could be significantly reduced using the proposed method in actual measurements as well as in simulations, and the *S*/*N* ratio was also improved to the same extent as in simulations. Even in the palm measurements, the initial sound pressure of the absorber reconstructed using the proposed method was lower than that reconstructed using only real sensor signals. These are also considered to be due to the same reason as in the simulation experiments.

In addition, the results for the proposed method using palm measurement data showed a tendency for meandering blood vessels to become slightly straighter. The reason for this is thought to be that only spherical and cylindrical absorbers were used in the training data. In other words, this is because the training data did not have information about the meandering absorber, so the signals at the virtual sensors were generated to approximate the cylindrical shape included in the training data. Therefore, we believe that this problem will be improved in the future by training the CNN using an absorber distribution that is closer to the actual blood vessel distribution.

Because the proposed method significantly reduced the computational cost during CNN training and execution, we were able to run the proposed method on a commercially available GPU (NVIDIA RTX A6000 was used in this study). Even if the proposed method is used, it takes a long time (about 1 month) to train our CNNs. Although CNN training takes a lot of time, the calculation time during measurement was approximately 7.5 s (using NVIDIA RTX A6000), so we think there is no problem in practical use. Furthermore, if CNNs are applied completely in parallel, the processing time will be 0.11 s (= 7.5 s/(34 + 21 + 13)), making real-time processing possible.

In this study, the proposed method was applied to a hemispherical array sensor based on the golden ratio and Fibonacci sequence, but the proposed method can also be applied to sensors such as a 2D grid array sensor on a plane. Furthermore, although the proposed method was applied to photoacoustic imaging in this study, it can also be applied to other measurements using similar hemispherical array sensor, such as ultrasound imaging. Therefore, we believe that the proposed method has a wide range of applications. On the other hand, even in systems that currently use dense array sensors, it is possible to reduce costs without reducing image quality using sparse array sensors and the proposed method.

One of the limitations of the proposed method is that it cannot be applied to two-dimensional array sensors where the sensors are not regularly arranged. For example, the proposed method cannot be applied to a 2D array sensor with randomly distributed sensors. In addition, although it was possible to generate virtual sensor signals by interpolating between real sensors, it is considered difficult to generate virtual sensor signals by extrapolating outside the real sensors.

## Conclusion

In this paper, we proposed a method to realize a virtual dense hemispherical array sensor by setting virtual sensors between the real sensors in three directions and generating virtual sensor signals using deep learning. We also applied the proposed method to simulations and human palm measurement data and confirmed that the proposed method can significantly reduce artifacts caused by sparse sensor density. The proposed method is a method that can reduce the calculation cost by performing 2D CNN processing in each of the three directions even for a hemispherical array sensor, and we showed that the proposed method can be executed on commercially available GPUs. Therefore, using our proposed method, high-quality photoacoustic images can be acquired without moving the hemispherical array sensor, and we will be able to observe the moving targets in real time. We hope that this will provide useful information for the development of medicine.

In the future, we plan to improve the accuracy of image reconstruction for clinical data by creating CNN training data based on actual blood vessel shapes. In addition, although we used a CNN with a simple structure in this study, we plan to investigate CNNs with deeper and more complex structures that can perform highly accurate estimation, and optimize each parameter related to CNNs. We also want to examine the possibility of real-time measurement of photoacoustic imaging in clinical applications.

### Supplementary Information

Below is the link to the electronic supplementary material.

Supplementary file1 (DOCX 57 KB)

## Data Availability

The data that support the findings of this study are not openly available because it contains commercially sensitive information.
