# Supplementary material for: Artifact reduction in photoacoustic images by generating virtual dense array sensor from hemispheric sparse array sensor using deep learning
Source: J Med Ultrason (2001). 2024 Mar 14;51(2):169–83. doi: 10.1007/s10396-024-01413-3 (PMC11098876; doi:10.1007/s10396-024-01413-3)
Supplement: Supplementary file 1 — Supplementary file1 (DOCX 57 KB) [file 10396_2024_1413_MOESM1_ESM.docx]

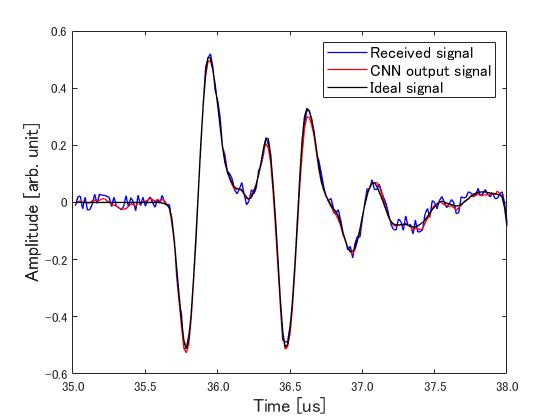


Fig. S1 Example of comparison between received signal and CNN output signal in a real sensor


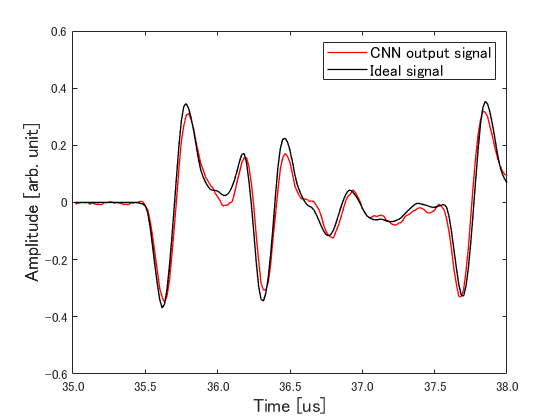


Fig. S2 Example of comparison between CNN output signal and ideal signal in a virtual sensor
